# Molecular Assessment Using the MASTDISCS® Combi D72C Set for the Phenotypic Detection of Extended-Spectrum Beta-Lactamases, AmpC Beta-Lactamases, and Carbapenemase Enzymes in Escherichia coli and Klebsiella pneumoniae

**DOI:** 10.7759/cureus.77269

**Published:** 2025-01-11

**Authors:** Sayran H Haji, Aryan R Ganjo, Sazan Moffaq Abdulaziz, Zheen A Abdullah, Sakar B Smail

**Affiliations:** 1 Department of Clinical Analysis, College of Pharmacy, Hawler Medical University, Erbil, IRQ; 2 Department of Medical Analysis, Faculty of Applied Science, Tishk International University, Erbil, IRQ; 3 Department of Medical Laboratory Technology, Erbil Technical Health and Medical College, Erbil Polytechnic University, Erbil, IRQ; 4 Department of Microbiology, Par Hospital, Erbil, IRQ

**Keywords:** ampc beta-lactamases, bata-lactam resistance, carbapenemase, esbl genes, escherichia coli, klebsiella pneumoniae

## Abstract

Beta-lactam resistance poses a global issue and a considerable challenge to effective antimicrobial therapy. The study aimed to ascertain the phenotypic and genotype traits of carbapenemase, extended-spectrum beta (β)-lactamases (ESBL), and AmpC β-lactamase-producing isolates collected from hospitals. A range of clinical samples consisted of 63 *Escherichia coli* *(E. coli*) and 30 *Klebsiella pneumoniae (K. pneumoniae)* isolates. Phenotypic characterization was carried out utilizing the MASTDISCS® Combi ESBL, AmpC, and carbapenemase detection set-D72C (Mast Group Ltd, Bootle, United Kingdom). Molecular assays were used to detect carbapenemase, ESBL, and AmpC genes. Both *E. coli* and *K. pneumoniae* clinical isolates exhibited noticeably enhanced resistance to β-lactam antibiotics. MASTDISCS® Combi D72C phenotype detection tests revealed that 57 (90.6%) *E. coli* and 30 (100%) *K. pneumoniae* isolates produced ESBL and AmpC enzymes, with evidence of carbapenemase activity. The majority of isolates had at least one β-lactamase-related gene. Based on molecular findings, the majority of ESBL-producing isolates in both pathogens had 17 (56.6%) of the bla_CTX-M_ gene in *K. pneumoniae* and 16 (53.3%) of the bla_SHV_ gene in both pathogens. The AmpC-associated genes, both bla_CMY1,_ and bla_CMY2_, were exposed in five (16.6%) *K. pneumoniae* isolates and nine (30%) and 10 (33.3%) among *E. coli,* respectively. In terms of the carbapenemase gene, bla_OXA_ was the most prevalent gene, appearing in 20 (66.6%) of the two pathogens.This study demonstrated that *K. pneumoniae *and* E. coli *that produceβ-lactamases have emergedas pathogens linked to infections in healthcare settings. Accurate identification of β-lactamase-producing bacterial pathogens is essential for patient treatment. We observed co-expression of AmpC, carbapenemase, and ESBL genes in most isolates, indicating a need to implement modern plans against these pathogens.

## Introduction

The emergence and spread of resistant strains to antimicrobial agents pose a significant global public health challenge. Primarily, resistance amongst Gram-negative pathogens, particularly those associated with the family of Enterobacteriaceae, *Escherichia coli* (*E. coli*) and *Klebsiella pneumoniae* (*K. pneumoniae*), is typified by their robust drug resistance [1]. In both hospitalized and outpatient settings, it is a substantial contributor to the development of a number of diseases, including pneumonia, sepsis, urinary tract infections (UTIs), and soft tissue infections [2].

Resistance to beta (β)-lactam compounds often emerges from multiple mechanisms, including the synthesis of enzymes that break down drugs, changes to the drug target (such as penicillin-binding proteins), decreased membrane permeability, and efflux pumps [3, 4]. Antibiotic overuse promotes the evolution of microorganisms that are resistant to many drugs. Infectious diseases caused by these strains are difficult to treat and impose significant financial burdens on healthcare systems and public health [5]. However, with the dramatic increase in carbapenem usage, the emergence of carbapenem-resistant species has become a mounting public health problem [2]. The worldwide emergence of Enterobacteriaceae that produce extended-spectrum beta-lactamases (ESBL), AmpC beta-lactamase (AmpC), and carbapenemase is a major worry for public health, given the restricted treatment choices available and elevated death rate [6]. The carbapenem group of antibiotics is frequently utilized as a last resort in treating infections caused by multidrug-resistant bacteria.

However, the increased expression of other beta-lactamases, membrane impermeability, carbapenemase synthesis, or a combination of these processes is frequently associated with decreased susceptibility to the carbapenem group in this family [3, 6]. Numerous investigations conducted in the Kurdistan region have shown that strains of K. pneumoniae and E. coli exhibit conglomerate resistance mechanisms involving AmpC and carbapenemases, leading to the development of multidrug resistance [7, 8]. The MASTDISCS® Combi D72C (Mast Group Ltd, Bootle, United Kingdom) was evaluated for its ability to identify the incidence of ESBL enzyme and/or AmpC enzyme production (including inducible AmpC) in Enterobacterales, along with confirmation of inducible AmpC and carbapenem resistance status [9, 10].

Multiplex polymerase chain reaction (PCR) facilitates the detection of multiple genes in a single reaction and coexisting genes in a single isolate. An accurate and quick diagnosis of resistance genes can inform therapeutic options [11]. Since resistance has emerged as a public health concern, early identification of resistant isolates and efficient infection control methods are required to minimize further spread. Prioritizing infection prevention and control methods in all healthcare settings is essential for limiting the spread of these pathogens [2]. Continuous monitoring is required to identify any further emergence of isolates with this resistance mechanism so that suitable mitigation strategies can be implemented [12]. The present research aims to analyze the phenotype and genetic characterization of ESBL, carbapenemase, and AmpC-producing isolates of *E. coli* and *K. pneumoniae* taken from hospitals.

## Materials and methods

Study design and bacterial strains

A research study was done on laboratory records of the positive cultures of patients with different types of infections who were admitted to the hospital during a 10-month period (February 2023 to December 2023). A total of 93 *E. coli *and *K. pneumoniae *isolates were obtained from various clinical samples sourced from microbiological laboratories in hospitals within Erbil city, in the Kurdistan Region of Iraq. The recovered isolates were identified according to their morphological characteristics using microscopical examination [13]. For confirmation, the Vitek-2 automated system with the Antimicrobial Susceptibility Testing card for Gram-negative organisms (AST-GN card; bioMerieux Inc., Salt Lake City, UT, USA) was utilized in accordance with the manufacturer's instructions from the hospital microbiology laboratory.

Antimicrobial susceptibility testing for isolates

The susceptibility of all isolates was ascertained by utilizing the disc diffusion method against various antimicrobials. These included amoxicillin (30 μg), cefepime (30 μg), cefotaxime (30 μg), imipenem (10 μg), meropenem (10 μg), ciprofloxacin (5 μg), and amikacin (30 μg), which were applied on Mueller-Hinton agar plates. All antibiotic discs were sourced from Oxoid Limited (Basingstoke, United Kingdom). *Klebsiella pneumoniae* American Type Culture Collection (ATCC) 700603 and *E. coli *ATCC 25922 were used for quality control in the antimicrobial susceptibility tests. Bacterial strains were classified as resistant, intermediate, or susceptible according to their inhibition zones using the Clinical and Laboratory Standard Institute guidelines (CLSI) [14]. *Escherichia coli* ATCC 25,922 and *K. pneumoniae* ATCC 13882 were used as the quality control reference strains, respectively.

Phenotype screening of ESBL, AmpC, and carbapenemase enzymes

Initially, 32 isolates from *E. coli* and 30 *K. pneumoniae* were tested for β-lactamase production using a commercial combination disc assay, the MASTDISCS® Combi ESBL, AmpC, and carbapenemase detection Set-D72C (Mast Diagnostics, Bootle, UK), based on the disc diffusion technique, the isolates classified as ESBL producers, AmpC-inducible producers, AmpC non-inducible producers, ESBL and AmpC co-producers, and suspected carbapenemase producers [10]. The set includes six discs: cefpodoxime 10 μg (disc A), cefpodoxime 10 μg + ESβL inhibitor (disc B), cefpodoxime 10 μg + AmpC inhibitor (disc C), cefpodoxime 10 μg + ESβL inhibitor + AmpC inhibitor (disc D), cefpodoxime 10 μg + ESβL inhibitor + AmpC inducer (disc E), and a penem antibiotic (disc F). The MASTDISCS® Combi D72C procedure was carried out according to the manufacturer's instructions. The results were analyzed following the manufacturer's guidelines. *Escherichia coli* National Collection of Type Cultures (NCTC) 13351 (ESBL), *Enterobacter cloacae* (*E. cloacae*) NCTC 13405 (AmpC), and *K. pneumoniae* NCTC 13438 (suspected carbapenemase) were used as positive controls for phenotypic confirmatory tests, while *E. coli* ATCC 25922 was used as a negative control.

Polymerase chain reaction screening for β-lactamase genes

The ESBL-encoding genes (bla_CTX_, bla_TEM_, and bla_SHV_), AmpC-coding genes (bla_CMY1_, bla_CMY2_, bla_DHA_, and bla_FOX_), and carbapenemase-encoding genes (bla_IMP_, bla_NDM_, bla_VIM_, bla_KPC_, and bla_OXA_-48) in 30 *E. coli* and 30 *K. pneumoniae* samples were investigated using PCR employing a set of primers as shown in Table 1.

**Table 1:** Specialized primers to identify genes encoding beta (β)-lactamases Carbapenemase genes: IMP: imipenemase; VIM: vimentin; NDM: New Delhi metallo-beta-lactamase; OXA: oxacillinase; KPC: *Klebsiella pneumoniae* carbapenemase AmpC genes: AmpC beta-lactamases; CMY1: cytochrome C1; CMY2: cytochrome C2; DHA: DHA beta-lactamase; FOX: cefoxitin ESBL: extended-spectrum beta-lactamase genes: CTX: cefotaximase; SHV: sulfhydryl reagent variable; TEM: temoneira

Gene type	Target gene	Primer sequences (5 ' - 3 ')	Amplicon size (bp)	Reference
Carbapenemase	bla_IMP_	5'-GGAATAGAGTGGCTTA ACTCTC-3' 5'-GGTTTA ACAAAACAACCACC-3'	232	[15]
	bla_VIM_	5'-GATGGTGTTTGGTCGCATA-3' 5'-CGAATGCGCAGCACCAG-3'	390	
	bla_NDM_	5'-GGTTTGGCGATCTGGTTTTC-3' 5'-CGGAATGGCTCATCACGATC-3'	621	
	bla_OXA_	5'-GCGTGGTTAAGGATGAACAC-3' 5'-CATCAAGTTCAACCCAACCG-3'	438	
	bla_KPC_	5'-CGTCTAGTTCTGCTGTCTTG-3' 5'-CTTGTCATCCTTGTTAGGCG-3'	798	
AmpC	bla_CMY1_	5'-GCTGCTCAAGGAGCACAGGATCCCG-3' 5'-GGCACATTGACATAGGTGTGGTGCATG-3'	522	[16]
	bla_CMY2_	5'-ACTGGCCAGAACTGACAGGCAAA-3' 5'-GTTTTCTCCTGAACGTGGCTGGC-3'	466	
	bla_DHA_	5'-CTTTCACAGGTGTGCTGGGTGCG-3' 5'-CCGTACGCATACTGGCTTTGCGC-3'	403	
	bla_FOX_	5'- CATGGGGTATCAGGGAGATGC C-3' 5'- GCCGCTGCTCGCCCATCG-3'	218	
ESBL	bla_CTX_	5¢ATGTGCAGACCAGTAAGATGGC-3¢ 5¢- TGGGTAATAGTACCAGAACAGCGG-3¢	593	[17]
	bla_SHV_	5'-CTTTATCGGCCCTCACTCAA-3' 5'-AGGTGCTCATCATGGGAAAG-3'	237	
	bla_TEM_	5'-CGCCGCATACACTATTCTCAGAATGA-3' 5'-ACGCTCACCGGCTCCAGATTTAT-3'	445	

The genomic DNA extraction kit (DNAL and Scientific, Cat No. GG2001, Viogene Biotech Corp., New Taipei City, Taiwan). The reaction mixture comprised 1 μl of each primer (10 μM), 12.5 μl of Taq Green PCR Master Mix (2X) (Thermo Fisher Scientific Inc., Waltham, MA, USA), 1 μl of total DNA, and 9.5 μl of nuclease-free water. The PCR program proceeded as follows: initial denaturation at 94˚C for 10 min, 35 cycles of DNA denaturation at 94˚C for 30 sec, annealing at 54˚C for 30 sec, extension at 72˚C for 1 min, and a final elongation step at 72˚C for 7 min. The PCR products were visualized by performing electrophoresis on 1% agarose gels [15].

Statistical examination

GraphPad Prism (version 5; GraphPad Software, La Jolla, CA, USA) was used to analyze the data. A chi-square test was used to see whether there was a significant link between β-lactamase production and the various clinical samples. A p-value of < 0.05 was considered statistically significant.

## Results

Identification and antimicrobial susceptibility testing of bacterial strains

In this research, 63 were determined to be *E. coli* and 30 to be *K. pneumoniae*, using standard biochemical assay methods. Out of the collected samples, 46 (49.4%) *E. coli* isolates and 15 (16.1%) *K. pneumoniae* isolates were derived from urine samples, and seven (7.6%) isolates of *E. coli *and 12 (13%) isolates of *K. pneumoniae* were acquired from sputum samples. Collected pus samples using swabs yielded four (4.3%) *E. coli* and three (3.2%) *K. pneumoniae* isolates. Furthermore, six (6.4%) *E. coli* isolates were found in blood samples, even though the blood samples lacked *K. pneumoniae* isolates. In general, urine samples contained a large part of the isolates (61, 65.6%), with sputum samples accounting for the second-highest proportion (19, 20.4%). The results revealed that* E. coli* isolates showed high resistance to amoxicillin (58, 92%), cefepime (54, 85.7%), cefotaxime (50, 79.3%), ciprofloxacin (40, 63.4%), and amikacin (33, 52.3%). On the other hand, resistance to meropenem and imipenem has been observed in only 10 (15.8%) of *E. coli* isolates for both antibiotics. Furthermore, *K. pneumoniae* isolates exhibited significantly elevated levels of resistance as well, particularly against amoxicillin (27, 90%), cefotaxime (25, 83.3%), cefepime (24, 80%), ciprofloxacin (21, 70%), and amikacin (18, 60%). However, the resistance rates were relatively lower for imipenem (nine, 30%) and meropenem (five, 16.6%) in *K. pneumoniae* isolates. Statistical examination of the data indicated a considerable difference in the incidence of resistance to antibiotics in* E. coli* and *K. pneumoniae* strains that produce β-lactamase recovered from different types of samples (p < 0.0009) (Table 2).

**Table 2:** Antibiogram of beta (β)-lactamase-producing Escherichia coli and Klebsiella pneumoniae isolates p-value < 0.0009

Type and number of isolates	Amoxicillin	Cefepime	Cefotaxime	Imipenem	Meropenem	Amikacin	Ciprofloxacin
*Escherichia coli* 63 (67.7%)	58 (92%)	54 (85.7%)	50 (79.3%)	10 (15.8%)	10 (15.8%)	33 (52.3%)	40 (63.4%)
								Klebsiella pneumoniae 30 (32.3%)	27 (90%)	24 (80%)	25 (83.3%)	9 (30%)	5 (16.6%)	18 (60%)	21 (70%)
																Total no. 93 (100%)	85 (91.3%)	78 (83.8%)	75 (80.6%)	19 (20.4%)	15 (16.1%)	51 (54.8%)	61 (65.5%)

Phenotype analysis of ESBLs, AmpC, and carbapenemase production

The D72C is a six-disc system designed to detect resistance in Enterobacteriaceae, which includes ESBL-positive strains, AmpC-positive strains (derepressed/hyperproduced, plasmid-mediated, and inducible), co-production of AmpC and ESBL enzymes, and screening for the production of carbapenemase enzymes. This test utilizes a combination disc set that comprises cefpodoxime, various inhibitors, and a carbapenem antibiotic. These components work synergistically to provoke a reaction, the interpretation of which relies on the difference in zone sizes for each disc.

**Figure 1:**
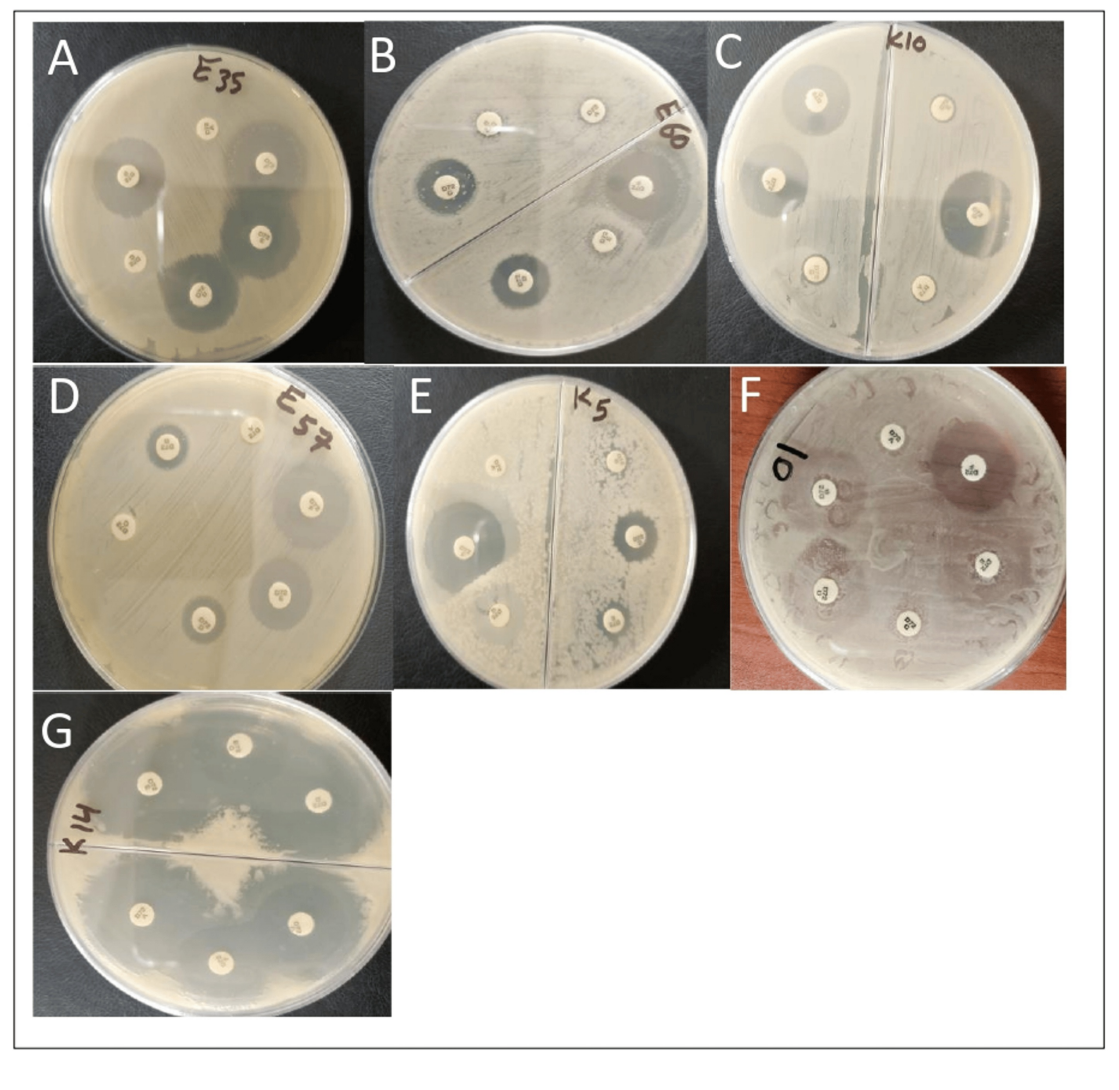
Phenotype detection results of extended-spectrum beta-lactamases (ESBL), AmpC beta-lactamases, and carbapenemase production by Escherichia coli and Klebsiella pneumoniae isolates detected by MASTDISCS Combi D72C tests A. ESBL; B. AmpC; C. inducible AmpC; D. ESBL and AmpC; E. suspected carbapenemase; F. Suspected carbapenemase co-producer; G. negative result

The results of the MASTDISCS® Combi D72C phenotype detection tests indicated that 90.6% (29 of 32) *E. coli *isolates and 100% (30 of 30) *K. pneumoniae* isolates produced ESBL and AmpC enzymes, showing signs of carbapenemase activity. Among the tested isolates, 25% (eight out of 32) of *E. coli* and 16.6% (five out of 30) of *K. pneumoniae* were identified as ESBL producers. Additionally, 12.5% (four out of 32) of *E. coli* and 16.6% (five out of 30) of* K. pneumoniae* exhibited AmpC production. Within this group, 6.2% (two out of 32) of *E. coli* and 13.3% (four out of 30) of *K. pneumoniae* isolates were inducible AmpC producers. Furthermore, 18.7% (six out of 32) of *E. coli* and 13.3% (four out of 30) of *K. pneumoniae* isolates were identified as co-producers of ESBL and AmpC enzymes. Additionally, 15.6% (five out of 32) of *E. coli *and 23.3% (seven out of 30) of *K. pneumoniae* isolates showed indications of suspected carbapenemase activity. For 12.5% (four out of 32) of *E. coli* and 16.6% (five out of 30) of* K. pneumoniae* isolates, there was evidence of suspected carbapenemase-co-producers (Figure 1). The statistical examination demonstrated a strong significance in the frequency of ESBL and AmpC enzymes, along with indications of carbapenemase activity, within β-lactamase-producing *E. coli* and *K. pneumoniae* isolates (p < 0.0001) (Table 3).

**Table 3:** Frequency of beta-lactamase-producing E. coli and K. pneumoniae isolates by phenotypic analysis ESBL: extended-spectrum beta-lactamase) genes; AmpC: AmpC beta-lactamases;* E. coli*: *Escherichia coli*;* K. pneumoniae*: *Klebsiella pneumoniae*

Bacteria	Tested isolates	ESBL production	AmpC beta-lactamase production		ESBL + AmpC co-producer	Carbapenemase production		Total no. of beta-lactamase-producing isolates
		ESBL	AmpC	Inducible AmpC		Suspected carbapenemase	Suspected carbapenemase co-producer	
E. coli	32	8( 25%)	4 (12.5%)	2 (6.2%)	6 (18.7%)	5 (15.6%)	4 (12.5%)	29 (90.6%)
K. pneumoniae	30	5 (16.6%)	5 (16.6%)	4 (13.3%)	4 (13.3%)	7 (23.3%)	5 (16.6%)	30 (100%)

In consequence, drawing from the PCR findings, it may be concluded that the MASTDISCS® Combi D72C test accurately identified β-lactamase production in 100% of *K. pneumoniae* and 90% of *E. coli* isolates, irrespective of the specific β-lactamase types present.

Polymerase chain reaction analysis of ESBLs, AmpC, and carbapenemase genes

**Figure 2:**
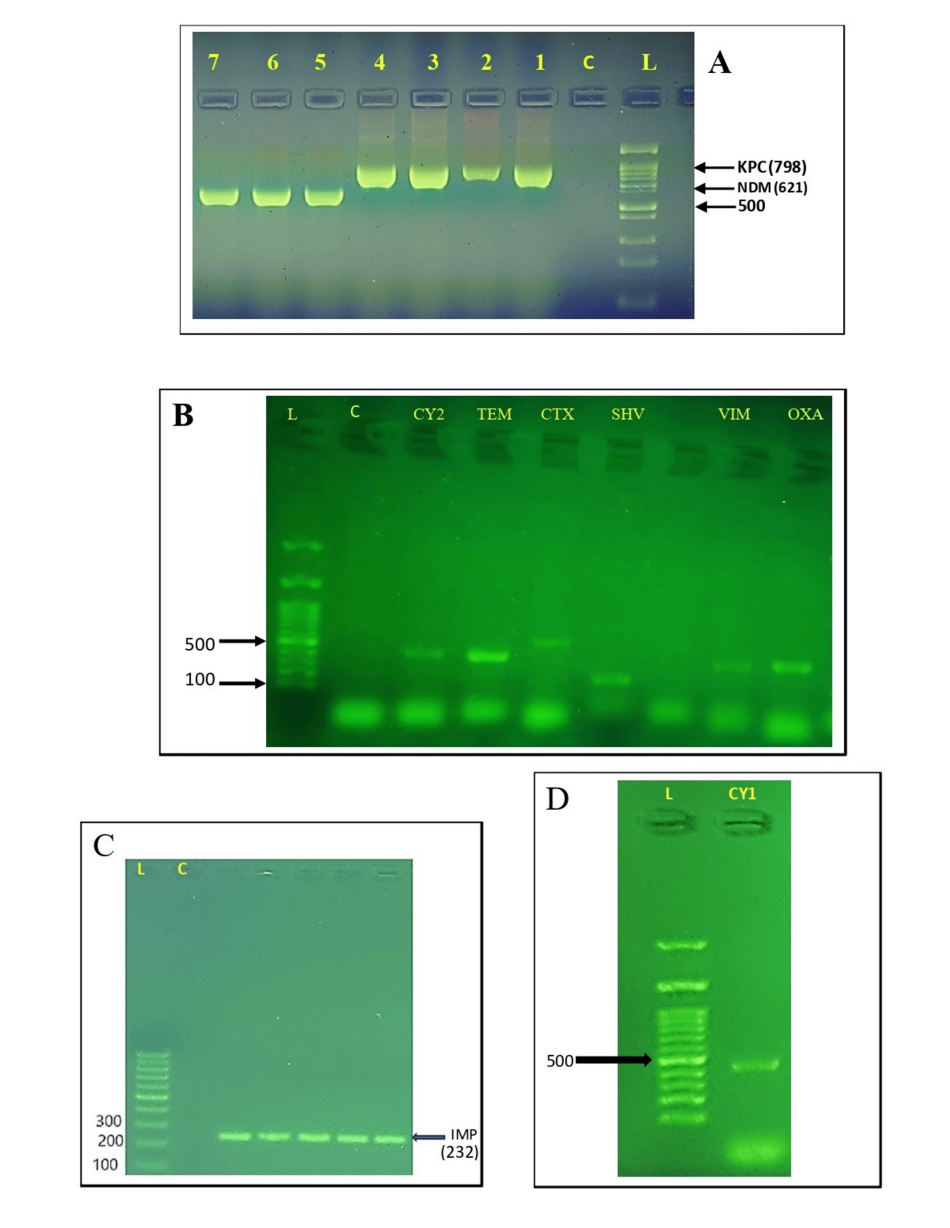
The results of the polymerase chain reaction (PCR) assay used for detecting beta (β)-lactamase genes Lane M represents a 1-kb DNA ladder, and lane C serves as a negative control. A. The gel electrophoresis displayed the presence of carbapenemase genes bla_KPC_ and bla_NDM_. B. Gel electrophoresis revealed the presence of various gene amplicons, including bla_CMY2_ (466 bp) for AmpC gene, and ESBL genes bla_TEM_ (445 bp), bla_CTX_ (593 bp), and bla_SHV_ (237 bp). Additionally, bla_VIM_ (390 bp) as well as bla_OXA-48_ (438 bp) carbapenemase genes were also detected. C. The gel electrophoresis specifically showed the presence of the carbapenemase gene bla_IMP_. D. The gel electrophoresis indicated the presence of the AmpC gene amplicon bla_CMY1_ (522 bp).

Molecular techniques demonstrated that 30 (100%) *K. pneumoniae* isolates examined carried multiple β-lactamase-related genes. These isolates' ESBL genes are in the following distribution: 56.6% harbored bla_CTX_, 53.3% had bla_SHV_, and 26.6% possessed bla_TEM_. Notably, bla_CTX_ was more prevalent in *K. pneumoniae* isolates that express ESBL, as depicted in Table 4 and Figure 2.

**Table 4:** Distribution of beta (β)-lactamase genes in Escherichia coli (E. coli) and Klebsiella pneumoniae (K. pneumoniae) isolates p-value < 0.0186 ESBL: extended-spectrum beta-lactamase genes: CTX: cefotaximase; SHV: sulfhydryl reagent variable; TEM: temoneira AmpC genes: AmpC beta-lactamases; CMY1: cytochrome C1; CMY2: cytochrome C2 Carbapenemase genes: IMP: imipenemase; VIM: vimentin; NDM: New Delhi metallo-beta-lactamase; OXA: oxacillinase; KPC: *Klebsiella pneumoniae* carbapenemase

Pathogens	Tested isolates no.	bla_CTX_ no. (%)	bla_SHV_ no. (%)	bla_ TEM_ no. (%)	bla_ CMY1_ no. (%)	bla_ CMY2_ no. (%)	bla_KPC_ no. (%)	bla_OXA_ no. (%)	bla_IMP_ no. (%)	bla_VIM_ no. (%)	bla_ NDM_ no. (%)
E. coli	30	12	16	10	9	10	5	20	5	9	8
		(40%)	(53.3%)	(33.3%)	(30%)	(33.3%)	(16.6%)	(66.6%)	(16.6%)	(30%)	(26.6%)
K. pneumoniae	30	17	16	8	5	5	7	20	3	16	8
		(56.6%)	(53.3%)	(26.6%)	(16.6%)	(16.6%)	(23.3%)	(66.6%)	(10%))	(53.3%)	(26.6%)

Similarly, for *E. coli* isolates, molecular methods showed that 28 (93.3%) of them carried two or more β-lactamase-related genes. Following are the* E. coli *clinical isolates' ESBL gene frequencies: 53.3% displayed the gene bla_SHV_, 40% exhibited the bla_CTX_, and 33.3% confirmed the existence of the bla_TEM_. In *E. coli* there was a higher incidence of bla_SHV_ among ESBL genes. Among *K. pneumoniae* isolates, AmpC-associated genes, namely bla_CMY1_ and bla_CMY2_, were found in approximately 16.6% of cases. The distribution percentages of bla_CMY1_ and bla*CMY2* among *E. coli* were 30% and 33.3%, respectively. Notably, all strains of *E. coli* and *K. pneumoniae* that were inspected were negative for bla_DHA_ and bla_FOX_ testing (Figure 2). The following prevalence rates were found for the genes linked to carbapenemase in the *E. coli* isolates: bla_OXA-48_ at 66.6%, bla_VIM_ at 30%, bla_NDM_ at 26.6%, bla_KPC_, and bla_IMP_ at 16.6%. For carbapenemase-positive *K. pneumoniae* isolates, the incidence of the respective genes was as follows: bla_OXA-48_ in 66.6%, bla_VIM_ in 53.3%, bla_NDM_ in 26.6%, bla_KPC_ in 23.3%, and bla_IMP_ in 10% of the isolates. Furthermore, statistical analysis indicated a notable variation in the frequency of β-lactamase-related genes among *E. coli* and *K. pneumoniae* isolates (p < 0.0186) (Table 4).

## Discussion

The global rise in antibiotic usage is resulting in the proliferation of resistant microorganisms, posing a significant and escalating healthcare challenge worldwide [3]. Our findings revealed a higher occurrence of resistance to commonly used antibiotics, consistent with the research conducted by others [6, 18]. Beta-lactam antibiotics are frequently administered to treat bacterial infections; however, the rise of resistance to these medications is increasingly worrisome. Various studies conducted in Iran and Egypt reported different rates of ESBL-producing isolates, with 40% of *K. pneumoniae* and 35.4% of *E. coli* isolates being ESBL producers in Iran [1], while in Egypt, the rates were higher at 84.4% for *E. coli *and 82.9% for *K. pneumoniae* [3]. The wide variation in prevalence across these studies may be attributed to differences in the types of specimens collected from various sources and inadequate antibiotic therapy, which could have contributed to the increased prevalence of ESBL [5]. The main reasons for the rise in ESBLs were found to be the widespread use of third-generation cephalosporins and the lack of routine testing on isolation of ESBL-producing strains in patients admitted to hospitals [19]. Regarding AmpC enzyme production, the MASTDISCS® Combi D72C phenotypic test identified 12.5% *E. coli *and 16.6% *K. pneumoniae* isolates that generated AmpC. Additionally, 6.2% of *E. coli* and 13.3% of *K. pneumoniae* isolates were found to have inducible AmpC production. The outcomes of this research closely resembled those of a prior study carried out in Egypt, which showed a lower prevalence (11.8%) of the AmpC enzyme among Enterobacteriaceae using a boronic acid test [20]. While higher percentages of AmpC-producing isolates were found in other countries, such as Iran, where 20% of *E. coli* isolates and 9.2% of *K. pneumoniae* were AmpC-producing [1]. In Egypt, the examined* E. coli* and *K. pneumoniae* isolates had a significant amount of AmpC (84.4% and 82.9%, respectively) [3]. In another investigation, employing an ESBL and MAST D72C AmpC detection kit, 10.2% of the 235 fresh vegetable samples were found to contain isolates of AmpC-producing *E. cloacae* [10]. A notable observation in this study is that many ESBL-positive isolates also produced AmpC, and some isolates produced ESBL and AmpC enzymes simultaneously, with 18.7% *E. coli* and 13.3% *K. pneumoniae* being co-producers of both beta-lactamases. This co-production was mentioned in numerous other studies as well [1, 3, 20, 21]. In a study conducted in Azerbaijan investigating multidrug-resistant Enterobacteriaceae that developed β-lactamase, 4.6% of the isolates were found to have hyperproduction of AmpC [22]. Regarding carbapenemase production detection using the MASTDISCS® Combi D72C phenotyping method, approximately 15.6% and 12.5% of the *E. coli* isolates and 23.3% and 16.6% of the *K. pneumoniae* isolates were identified as carbapenemase producers. In another study by Haji et al., the Carba Plus assay (D73C) identified 59% of Gram-negative bacilli isolates as carbapenemase producers [7]. Similarly, phenotypic detection using a modified Hodge test by Kazemian et al. showed that 27.7% of *E. coli *isolates and 43.3% of *K. pneumoniae* isolates were carbapenemase producers [1]. The MASTDISCS® Combi D72C showed high accuracy in identifying β-lactamase producers, correctly identifying 93.3% of *E. coli* isolates and 100% of *K. pneumoniae* isolates with β-lactamase activity. Numerous studies conducted in other countries have consistently identified blaCTX-M1 [3, 11, 23] as the gene that encodes β-lactamases most often in ESBL-producing *E. coli* and *K. pneumoniae*, as well as in other Enterobacteriaceae [24]. The bla_CTX-M_ exhibits the potential for horizontal transfer through various moving genetic components. Another investigation into hospital-acquired infections revealed that the bla_CTX-M_ gene was discovered to be present in about 21.5% of *E. coli* isolated, in contrast, along with bla_TEM_, 16.9% of the isolates contained bla_SHV_. For *K. pneumoniae* strains [1]. The predominant AmpC variants were bla_CMY-1_ and bla_CMY-2_, with bla_DHA_ and bla_FOX_ not being detected. These findings align with the global distribution of AmpC subtypes and are in line with earlier research conducted in Iran [1, 25] and Egypt [3]. In Iran, previous data showed the occurrence of bla_DHA_, bla_CMY_, bla_FOX_, and bla_MOX_ in clinical *E. coli *isolates [26]. Regarding carbapenemase genes, the most common type was bla_OXA-48_, followed by bla_VIM_ and bla_NDM_ among *E. coli* and *K. pneumoniae* isolates; bla_KPC_ and bla_IMP_ were less frequently detected. Notably, the commonness of bla_OXA-48_ was consistent with other studies conducted in different nations [1, 27, 28]. Also, in a study from Iran, bla_OXA-48_ was the predominant carbapenemase gene, detected in 58.3% of isolates, followed by bla_IMP_ (41.7%) and bla_NDM_ (8.3%). None of the isolates harbored bla_VIM_ and bla_KPC_ genes [29]. In another study, the bla_CTX-M_ was the predominant (44%) gene, followed by bla_TEM_ (24%) and bla_SHV_ (8%), which was much lower than reported in the current study [30]. The current research discovered a significant proportion of isolates co-producing multiple genes simultaneously among β-lactamase-producing *E. coli* (93.3%) and *K. pneumoniae* (100%). The coexistence of ESBL, AmpC, and metallo-β-lactamases has also been documented in various other nations [1, 3, 11]. Additionally, in Enterobacteriaceae, there is a widespread occurrence of resistance to carbapenems, which is associated with AmpC hyper-production and diminished permeability (porin loss) or ESBL [8].

Limitations of the study

There are some limitations to the current investigation. The study's sample size may have an impact on the finding's accuracy and generalizability because it was carried out in a small area. While the MASTDISCS® Combi D72C test is effective for detecting β-lactamase production, it may not identify all variants of these enzymes. Some resistant strains may produce enzymes that are not detected by this specific phenotypic method, potentially leading to an underestimation of resistance rates. Although molecular assays were used to detect specific resistance genes, the study did not explore the mechanisms of gene transfer or the genetic context of these resistance genes, which are important for understanding the epidemiology of resistance. These limitations highlight the need for further research to validate the findings and explore the broader implications of β-lactam resistance in clinical settings.

## Conclusions

Healthcare-associated infection caused by beta-lactamase-producing *E. coli* and *K. pneumoniae* is a major concern. The recovered clinical isolates from the current study demonstrated notable resistance to antibiotics. The molecular analysis revealed that the bla_CTX-M_ gene was prevalent. For AmpC-associated genes, bla_CMY1_ and bla_CMY2_ were detected in 16.6% of *K. pneumoniae* isolates and 30% and 33.3% among *E. coli* isolates, respectively. The most common carbapenemase gene identified was bla_OXA_, indicating a significant concern for treatment options. It has been concluded that the MASTDISCS® Combi D72C test accurately identified β-lactamase production in 100% of *K. pneumoniae* and 90% of *E. coli* isolates, irrespective of the specific β-lactamase types present. The most prevalent gene was ESBL genes in both *E. coli* and *K. pneumoniae*. The use of MASTDISCS® Combi D72C in routine *E. coli* and *K. pneumoniae* sensitivity tests could assist as a valuable early indicator for β-lactamase-producing isolates. An urgent need for improved antibiotic stewardship and robust infection control practices to mitigate the public health threat posed by ESBL, AmpC, and carbapenemase-producing *E. coli *and *K. pneumoniae*. These measures are essential to preserve the effectiveness of existing antibiotics and protect patient health in healthcare settings.
